# A mosaic genetic screen for novel mutations affecting *Drosophila *neuroblast divisions

**DOI:** 10.1186/1471-2156-7-33

**Published:** 2006-06-02

**Authors:** Cathy Slack, W Gregory Somers, Rita Sousa-Nunes, William Chia, Paul M Overton

**Affiliations:** 1 Department of Biology, University College London, Gower Street, London WC1E 6BT, UK (current address); 2 Temasek Lifesciences Laboratory, The National University of Singapore, 117604, Singapore; 3 MRC Centre for Developmental Neurobiology, King's College London, UK

## Abstract

**Background:**

The asymmetric segregation of determinants during cell division is a fundamental mechanism for generating cell fate diversity during development. In *Drosophila*, neural precursors (neuroblasts) divide in a stem cell-like manner generating a larger apical neuroblast and a smaller basal ganglion mother cell. The cell fate determinant Prospero and its adapter protein Miranda are asymmetrically localized to the basal cortex of the dividing neuroblast and segregated into the GMC upon cytokinesis. Previous screens to identify components of the asymmetric division machinery have concentrated on embryonic phenotypes. However, such screens are reaching saturation and are limited in that the maternal contribution of many genes can mask the effects of zygotic loss of function, and other approaches will be necessary to identify further genes involved in neuroblast asymmetric division.

**Results:**

We have performed a genetic screen in the third instar larval brain using the basal localization of Miranda as a marker for neuroblast asymmetry. In addition to the examination of pupal lethal mutations, we have employed the MARCM (Mosaic Analysis with a Repressible Cell Marker) system to generate postembryonic clones of mutations with an early lethal phase. We have screened a total of 2,300 mutagenized chromosomes and isolated alleles affecting cell fate, the localization of basal determinants or the orientation of the mitotic spindle. We have also identified a number of complementation groups exhibiting defects in cell cycle progression and cytokinesis, including both novel genes and new alleles of known components of these processes.

**Conclusion:**

We have identified four mutations which affect the process of neuroblast asymmetric division. One of these, mapping to the *imaginal discs arrested *locus, suggests a novel role for the anaphase promoting complex/cyclosome (APC/C) in the targeting of determinants to the basal cortex. The identification and analysis of the remaining mutations will further advance our understanding of the process of asymmetric cell division. We have also isolated a number of mutations affecting cell division which will complement the functional genomics approaches to this process being employed by other laboratories. Taken together, these results demonstrate the value of mosaic screens in the identification of genes involved in neuroblast division.

## Background

The development of the nervous system of higher organisms requires the generation of an extraordinary cellular diversity. One mechanism by which this diversity can be established is the segregation of cell fate determinants to one specific daughter during cell division thereby generating progeny with different cellular identities. Neuroblasts, the *Drosophila *neural progenitors, have served as one of the major models for studying asymmetric division (reviewed in [1]). Neuroblasts divide along an apical-basal axis, utilizing apical cues inherited from the neuroectoderm out of which they delaminate [2,3], to generate daughter cells with distinct identities. The large apical daughter cell retains its neuroblast identity and continues to divide while the small basal daughter cell, the ganglion mother cell (GMC), undergoes a single division to generate two postmitotic progeny of neuronal or glial identity.

The initial step in defining the asymmetry of neuroblast divisions is the establishment at the apical cortex of a multi-protein complex (reviewed in [4,5]) containing Inscuteable and two highly conserved signalling cassettes, the Par proteins – Bazooka (the *Drosophila *homologue of Par-3), Par-6 and atypical protein kinase C (DaPKC) – and the heterotrimeric G protein subunit Gαi together with the guanine nucleotide dissociation inhibitors Partner of Inscuteable (Pins) and Locomotion defects (Loco). The apical complex has several important functions during neuroblast asymmetric division including the correct orientation of the mitotic spindle along the apical-basal axis of the cell, the displacement of the spindle towards the basal cortex [6,7] and the establishment of a difference in spindle length between its apical and basal halves at anaphase [6,8]. This gives rise to a dramatic size asymmetry between daughter cells, with a small basal GMC budding from a large apical neuroblast. The apical complex is also essential for directing the localization of cell fate determinants to the neuroblast basal cortex. Phosphorylation of Lethal giant larvae (Lgl) by DaPKC appears to lead to the activation of Myosin II and the exclusion of Miranda from the apical cortex [9-11]. Myosin VI (Jaguar) is also required for basal localization of Miranda [12], although the mechanisms by which Miranda is transported and/or anchored to the basal cortex remain unknown.

Miranda functions as an adapter protein, localizing Staufen and Prospero (Pros) to the basal cortex [13-15]. Staufen, an RNA-binding protein required in the oocyte to localize *bicoid *mRNA [16], is employed in the neuroblast to anchor *pros *mRNA basally [17-19]. The segregation into the basal daughter cell of the homeodomain protein Prospero and its mRNA is the critical step in establishing GMC identity [20-22]. In the GMC, Pros translocates to the nucleus where it regulates gene expression, directing a drastic change in cellular identity [23-25].

Several molecules known to be involved in asymmetric neuroblast division have been identified in zygotic loss of function screens looking for embryonic phenotypes but the major limitation of this method is that maternal contribution of mRNA will mask the effects of the loss of many genes during embryogenesis. In support of this idea, animals lacking zygotic *Gαi*, *Pins *or *Loco *– three components of the apical complex identified biochemically or by a candidate gene approach – are viable, albeit with locomotion defects, and fertile, indicating that alleles of these genes would not have been found in a zygotic loss of function genetic screen [26-29].

A systematic germline clone screen might be an effective way to identify new components of the asymmetric cell division machinery. However, components such as Myosin II and Jaguar are required during oogenesis and do not give rise to fertilized eggs in germline clones [30,31], and we considered that such a screen would miss a number of the genes involved in neuroblast division.

To minimize the complications of maternal contribution or requirement of components of the asymmetry machinery in oogenesis, we decided to avoid embryonic neuroblasts entirely and switch to examining asymmetric division in third instar larval neuroblasts, which are known to employ most of the machinery used in embryos in an analogous manner [32]. Analysis of mutations in third instar larvae is possible directly where homozygous mutant animals survive until this stage and the phenotype of *pins *mutants has been examined in this way [33]. However, the majority of mutations in genes with important roles during development – including many of the molecules known to be required for neuroblast asymmetric division – are lethal before the third larval instar, and cannot be examined in this way. The classic method to circumvent early lethality in *Drosophila *is to generate postembryonic clones of cells homozygous for the mutation of interest, and we have used a variation of such a strategy in which clones are positively marked by the expression of GFP: the MARCM system (Mosaic Analysis with a Repressible Cell Marker; [34]), which has been used previously in a screen for phenotypes in mushroom body clones [35]. Core to the MARCM system is the use of the yeast GAL80 repressor, which blocks transcriptional activation by GAL4. Generation of somatic clones lacking GAL80 and homozygous for a mutation of interest in animals expressing GAL4 allows expression of UAS-*CD8::GFP *only within the clone. We considered an additional advantage to the MARCM approach. A number of molecules which have recently been implicated in neuroblast asymmetry, including Lgl, Myosin II, Myosin VI and Cdc2 [36], as well as Rab11 and Sec15 – components of the vesicular trafficking machinery [37,38] – have highlighted the importance of the general cellular machinery in asymmetric cell division. A clonal screen, therefore, has the advantage that mutations in components of the cell division machinery can be identified by a lack or an excess of proliferation within clones, which can then be examined with regard to markers of asymmetric cell division.

In this study, we describe the results of a MARCM screen on chromosome arm 3L, together with a screen of pupal lethal and semi-lethal mutations on chromosome 3. We identified 78 mutations affecting neuroblast division that fall into 48 complementation groups. The majority of these represent genes required for cell division, 12 of which correspond to previously described loci, and several of which also appear to have effects on asymmetric cell division. Although the bulk of the cell division mutants isolated in our screen do not have clear polarity phenotypes, we have deficiency mapped many of them to small genomic regions, reasoning that this would complement several RNAi-based screens currently being conducted to look for genes involved in cell division (for example [39]). In addition to the cell division complementation groups, we found new loci involved in neuroblast asymmetric division, with phenotypes affecting spindle orientation, localization of basal determinants and neuroblast cell fate.

## Results

### Screen design and overview

**Figure 1:**
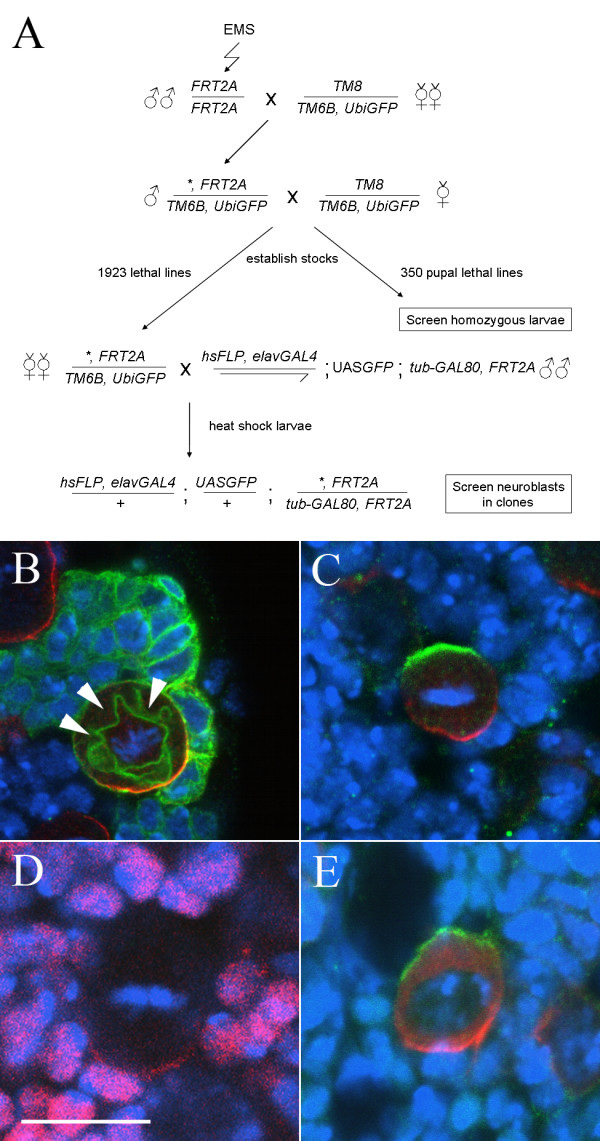
**A screen for asymmetric division mutants in third instar larval neuroblasts**. (A) Crossing scheme used to establish mutant lines and generate MARCM clones of mutations lethal before the third instar stage. The asterisk indicates an EMS-induced mutation. (B-E) Expression of asymmetric cell division markers in third instar larval neuroblasts. DNA staining (blue); Miranda (red, B, C, E); Prospero (Red, D); CD8::GFP (green, B); Inscuteable (green, C, E). Bar: 10 μm. Wild type clones (B) contain a single large neuroblast, with a crescent of Miranda at metaphase, and a cluster of smaller Miranda negative progeny. Note that the CD8::GFP membrane marker outlines the mitotic spindle (arrowheads). (C) The apical and basal markers Inscuteable and Miranda are localized to opposite poles of third instar metaphase neuroblasts, while crescents of Prospero are seen in most neuroblasts (D); note that the neuroblast Pros staining is weaker than the nuclear staining in neighbouring cells. In contrast to embryonic neuroblasts, Miranda does not colocalize with Inscuteable in early prophase but rather is found both in the cytosol and in a cortical crescent at the opposite pole of the cell to Inscuteable (E).

To systematically identify genes involved in neuroblast division, while minimising the effects of maternal contribution, we have used a mosaic approach, employing the MARCM system [34]. To confirm the suitability of this system, we first generated clones of a wild type chromosome and examined the expression and localization of proteins known to be asymmetrically distributed in metaphase neuroblasts. As expected, within each clone we see a single large neuroblast with a variable number of smaller progeny labeled with CD8::GFP under the control of *elavGAL4*^*C155*^, which directs expression in neuroblasts as well as neuroblast progeny (Figure 1B). As we anticipated from previous reports [32], the localization of known components of the asymmetric cell division machinery in metaphase neuroblasts appears to follow the model established from studies in embryos. Although larval neuroblasts do not have a clear apical-basal polarity with respect to the overlying epithelium, such as that defined in embryonic neuroblasts, known apical components localize in a cortical crescent opposite a crescent of known basal components, with the metaphase plate aligned in between (Figure 1C). In contrast to previous findings [32], we also observe localization of Prospero to the basal cortex of >90% of metaphase neuroblasts (Figure 1D). We do observe one minor difference between larval neuroblasts and those of the embryo. In embryonic neuroblasts, the basal marker Miranda is first localized to the apical cortex in early prophase and then translocated to form a tight basal crescent at metaphase [14,15,19]. In larval neuroblasts we never observe co-localization of Miranda with Inscuteable. Rather, during early prophase Miranda is found both cytoplasmically and in a cortical crescent at the opposite pole of the cell to Inscuteable (Figure 1E and data not shown). These observations confirm the validity of this clonal screening approach for neuroblast phenotypes, and suggest that the same or a largely overlapping repertoire of molecules is employed in larval and embryonic neuroblasts. As localization of the basal components is downstream of correct apical complex function, we chose to use an antibody against Miranda [40], together with the orientation of the metaphase plate and relative cell size in neuroblast clones, as final readouts of neuroblast asymmetric division.

To generate mutant lines, male flies isogenic for a chromosome carrying an *FRT *insertion at 79D-F (*FRT2A*) were mutagenized with ethyl methanesulfonate (EMS) and stocks established carrying mutations balanced over *TM6B*, *Tb*. In total we generated 1923 stocks carrying mutations causing lethality before the wandering third instar larval stage, as assayed by the absence of *Tb*^+ ^larvae, and approximately 350 pupal-lethal and semi-lethal chromosomes.

The 350 pupal- and semi-lethal lines giving rise to third instar larvae homozygous for the mutant chromosome were screened by antibody staining of non-*Tb *larvae. The crossing scheme for generating somatic clones of the remaining 1923 mutant lines is given in figure 1A. Females from our mutant lines were mated to males carrying *tub-GAL80 *on the *FRT2A *chromosome together with *elavGAL4*^*C155*^, *hsp70Flp *and UAS-*CD8::GFP*. Induction of mitotic recombination by 37° heatshocks at first and second instar stages resulted in the generation of clones within neural lineages that were homozygous for mutations on 3L and positively marked by the expression of CD8::GFP. Clones were readily identifiable by examination of intact larvae under UV light revealing that the great majority of *Tb*^+ ^female third instar larvae contained mutant clones in the brain. Nervous systems from these larvae were dissected and screened by antibody staining against Miranda and GFP and confocal microscopy. At least three metaphase or anaphase neuroblasts in at least two brains were examined looking for alteration or absence of the basal Miranda crescent or any misorientation of the metaphase spindle.

#### Pupal lethal screen

**Figure 2:**
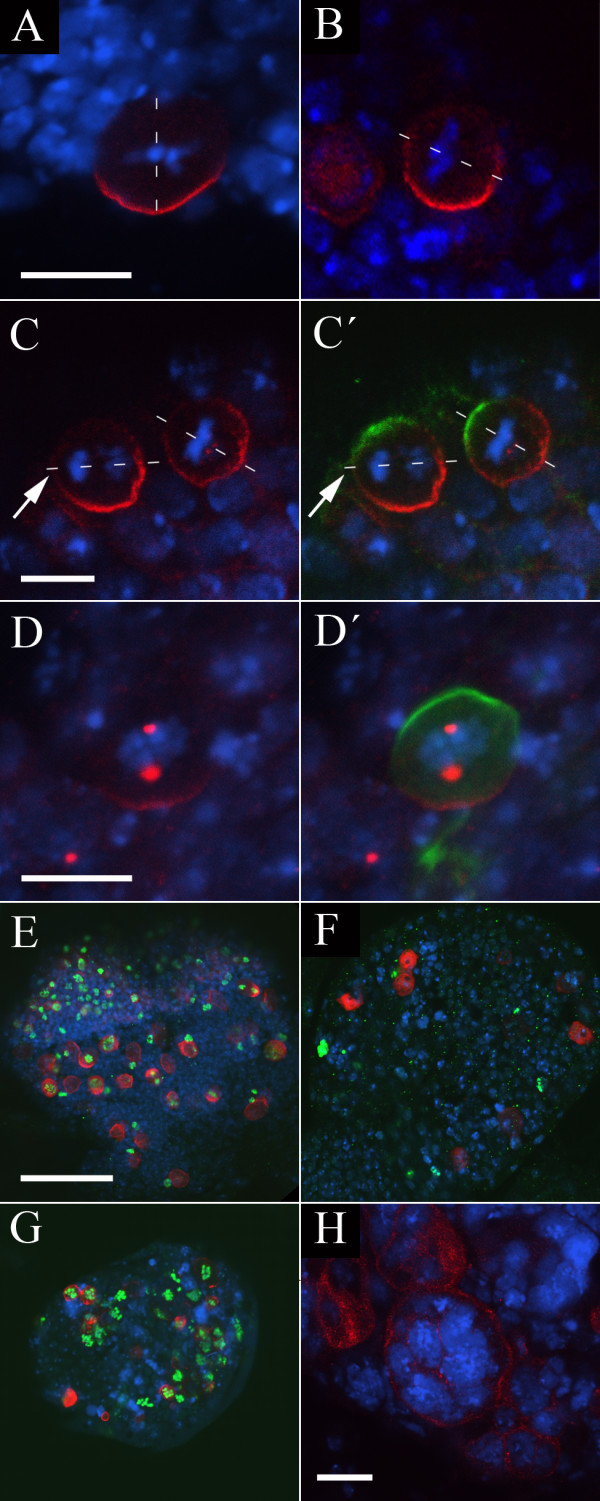
**Mutations identified in the pupal lethal screen**. (A-C') The *PL26 *mutation causes misorientation of the mitotic spindle in dividing neuroblasts. DNA staining (blue); Miranda (red); Inscuteable (green). The orientation of the mitotic spindle as judged by the orientation of the metaphase plate or anaphase chromosomes is indicated by a dotted white line; Bar: 10 μm. In wild type neuroblasts (A) the mitotic spindle is oriented perpendicular with respect to the Miranda crescent. In *PL26 *mutant larvae (B) the spindle is misoriented with respect to the Miranda crescent in 16% of neuroblasts (n = 75). (C, C') Cortical crescents of Inscuteable and Miranda are correctly localized to opposite poles of the cell in *PL26*. Note the misorientation of the spindle during anaphase in the cell to the left (arrows). (D, D') Miranda is mislocalized in *PL17 *neuroblasts. DNA staining (blue); Miranda (red); Inscuteable (green, D'). Bar: 10 μm. In mitotic *PL17 *neuroblasts, Miranda is localized to discrete cytoplasmic regions; in some neuroblasts Miranda crescents are also seen. Localization of the apical marker Inscuteable is not affected (D'). (E-G) The *PL13 *and *PL17 *mutations affect cell proliferation. DNA staining (blue); Miranda (red); Phospho-Histone H3 (green); Bar: 50 μm. In wild type optic lobes (E) 45% (n = 60) of neuroblasts stain with anti-PH3. In *PL13 *larvae (F) fewer neuroblasts are present and of these only 22% (n = 37) stain with anti-PH3. *PL17 *optic lobes (G) are reduced in size but 76% (n = 45) of neuroblasts stain with anti-PH3. (H) *IV61 *is allelic to *sticky*, and homozygous larval neuroblasts are large and have an increased DNA content. DNA staining (blue); Miranda (red); Bar: 20 μm.

We initially screened our collection of approximately 350 third chromosome pupal- and semi-lethal mutations for those that showed a neuroblast phenotype in the larval brain, scoring at least five dividing neuroblasts in at least two brains. We identified two lines with neuroblast asymmetry defects (Figure 2A–D; Table 1: Asymmetric cell division defects), and four lines with defects in neuroblast cell division. All of these lines cause lethality at pupal or pharate adult stages, and complementation testing between them indicated that these six mutants represent five separate complementation groups (Figure 2E–H; Table 1: Cell division defects).

**Table 1:** Mutations isolated. Summary of complementation analysis and phenotypic classes identified. Lines were placed into complementation groups following pairwise testing of all mutations identified. The gene affected is listed where known; otherwise the smallest deficiency or combination of deficiencies uncovering the mutation is shown together with the cytological region. Where we have only one allele in a complementation group which fails to complement multiple regions all lethal deficiencies are listed. Notes: ^*i*^Identified in our pupal lethal screen. ^*ii*^Mapped by recombination with rucuca chromosome followed by clonal analysis.

**Group**	**Alleles**	**Gene/lethal deficiency**	**Cytology**	**Further mapping data/Comments**
**Asymmetric cell division defects**				
**1**	PL26^*i*^	*Df(3R)p712*	84D4;85B6	No phenotype is observed in *PL26*/*Df(3R)p712 *hemizygotes
**6**	PL17^*i*^	*ida*		
**13**	J16	Not in deficiency kit	3L	Also carries a mutation in *polo*
**8**	D76	Not in deficiency kit	61F8;72D1^*ii*^	Lethality is caused by a mutation in *trio *which does not cause the Miranda phenotype
				
**Cell division defects**				
	*Proliferation defects*			
**2**	LVC73^*i*^	*Df(3L)GN24 *or *Df(3L)st-f13*	63F4;64C15 or 72C1;73A4	
**3**	PL13^*i*^	*Df(3R)WIN11 *or *Df(3R)Dr-rv1*	83E1;84A5 or 99A1;B11	
**6**	PL17^*i*^	*ida*		
**7**	A55, GL72	*small-minded*		
**8**	D76	*Df(3L)Ar14-8*	61C5;62A8	Lethality in this region is caused by a mutation in *trio*
**9**	A38, B10, B18	*Df(3L)Exel6112 *+ *Df(3L)ED4408*	66B5;66C5	
**10**	A57	*Df(3L)ZN47 *or *Df(3L)fz-GF3b*	64C;65C or 70C1;D5	Phenotype maps to 70C1;70D5 region^*ii*^
**11**	C10	*Df(3L)Ar14-8 *or *Df(3L)AC1*	61C5;62A8 or 67A2;D13	Phenotype maps to 67A2;67D13 region^*ii*^
**12**	E45	*makos*		
				
	*Chromosome separation defects*			
**14**	A9, A67, B14, H10, DL42	*Klp61F*		
**15**	G34	*Df(3l)ri-XT1*	77E2;78A4	Complements *Df(3L)*ED4861, Df*(3L)ME107*
**16**	C93	*separase*		Hemizogotes show multiple crescents of Miranda
				
	*Multinucleate cells*			
**5**	D97, CMV111^*i*^, IV61^*i*^	*sticky*		
**17**	C33	*pebble*		
**18**	A59, H2, GL22, C22	*Taf-4*		
**19**	A42	*Df(3L)ED4858 *+ *Df(3L)Exel6136*	77B2;77C1	
**20**	B27, H87	*Df(3L)BSC13 *+ *Df(3L)ED4408*	66B12;66C5	
**21**	C26, C36	*Df(3L)XDI98*	65A2;65E1	Complements *Df(3L)ZN47 *and *Df(3L)BSC27*
**22**	D7, CL89	*Df(3L)GN34 *+ *Df(3L)ED4341*	63F6;64A9	Complements *Df(3L)Exel6099*
**23**	D24	*Df(3L)Exel7253*	73D5;73E4	
**24**	D67, OL77	*Df(3L)ri-XT1*	77E2;78A4	Complements *Df(3L)ED4861*
**25**	D75	*Df(3L)R-G7 *or *Df(3L)vin7 *+ *Df(3L)eyg*^*C1 *^or *Df(3L)fz-M21 *+ *Df(3L)XG-5*	62B8;F5 or 69A4;B5 or 71C2;E5	
**26**	F58	*Df(3L)BSC33*	65E10;65F6	
**27**	GL45	*Df(3L)Exel6105*	64D1;64D6	
**28**	H67, J2	*Df(3L)Exel6087*	62A2;62A7	Complements *Df(3L)ED4238*
**29**	D40	7 lethal deficiencies in 4 lethal regions	63C2;F7 or 65F3;F6 or 66B8;C5 or 66E1;E6	
**30**	B55	Not in deficiency kit	3L	
**31**	E47, GL26	Not in deficiency kit	3L	
				
	*Vesicular/membrane defects*			
**32**	B44, C19, C62	*Aats-ile*		
**33**	A69	*Int6*		
**34**	D56	*neurexin*		Multinucleate cells are also observed with low frequency
**35**	E25, E55	*reptin*		
**36**	B11, O29	*Taf-6*		
**37**	A11, A572, A58, E80, O49	*Df(3L)X-21.2*	71F1;72A2	
**38**	A44	*Df(3L)GN24 *or *Df(3L)vin5 *+ *Df(3L)vin7 *or *Df(3L)fz-M21*	63F4;64C15 or 68C8;69A3 or 70D2;71E5	
**39**	OL61	*Df(3L)AC1*	67A2;67D13	
**40**	B29, H26	*Df(3L)ED4858*	76E1;76F1	Complements *Df(3L)ED229 *and *Df(3L)ED4861*
**41**	CMV45, ML72	*Df(3L)rdgC-co2*	77C6;77D1	Complements *Df(3L)ED4858 *and *Df(3L)Exel6136*
**42**	M7	*Df(3L)66C-G28 *or *Df(3L)rdgC-co2 *+ *Df(3L)ri-79c*	66B8;C10 or 77B;D1	
**43**	OL24	*Df(3L)X-21.2*	71F1;72A2	
**44**	CL62	*Df(3L)ED4858*	76D3;77C1	
**45**	F582, G82, ML72	*Df(3L)Exel6132 *+ *Df(3L)Exel9005*	74B2;74D2	ML72 is also allelic to CMV45 (group 41)
**46**	GL29	*Df(3L)ZP1 *or *Df(3L)ED218*	66A17;C5 or 71B1;E1	Phenotype maps to 71B1;71E1 region^*ii*^; complements *Df(3L)Exel6125*
**47**	A41, E50	not in deficiency kit	3L	
**48**	B57	n.d.	3L	
**4**	C79	n.d.	3L	

#### Clonal screen

We screened our collection of 1923 lethal chromosomes for phenotypes in MARCM neuroblast clones, looking particularly for defects in the formation and localization of the Miranda crescent but also more generally for clones exhibiting cell division phenotypes. We identified two lines with defects in neuroblast asymmetric division (Table 1: Asymmetric cell division defects), and a total of 77 mutations falling into 46 complementation groups affecting cell division (Table 1: Cell division defects).

An additional class of mutations failed to give rise to neuroblast containing clones, and we observed either no clones in these brains or clones of one or two neuronal cells, typical of neuron or GMC clones; we never observed clones larger than two cells not containing a neuroblast. Although in principle these small clones might reflect inappropriate neuroblast differentiation, the majority of these lines most likely carry cell lethal mutations, and were not examined further.

### Asymmetric cell division defects

#### PL26

In our pupal lethal screen, we identified a single mutation with a neuroblast polarity phenotype. In animals homozygous for the *PL26 *chromosome, we identified a misalignment of the mitotic spindle with respect to the Miranda crescent in a proportion of metaphase/anaphase neuroblasts. We scored misoriented spindles as those which form an angle of greater than 22.5° with the apical-basal axis of the cell, defined by the position of the Miranda crescent – in wild-type metaphase/anaphase neuroblasts we never see this degree of misorientation. In *PL26 *brains, we observed 12/75 neuroblasts with >22.5° misorientation compared to 0/63 for the *FRT2A *progenitor chromosome (Figure 2A, B), although it is presently unclear what proportion of these misoriented spindles will result in a failure to appropriately partition cell fate determinants into the GMC, and we have not investigated whether a telophase rescue of this misalignment – such as has been described in embryos [2,41] – might occur. We co-stained neuroblasts using an antibody against the *Inscuteable *protein, and found that the Miranda crescent forms opposite the Inscuteable crescent in all cases, but the metaphase plate is not correctly aligned with respect to the neuroblast apical-basal axis (Figure 2C). Clones homozygous for the left arm of this chromosome do not show any phenotype (not shown) but it is possible that this lack of phenotype reflects a perdurance of the wild type gene product rather than necessarily localizing the *PL26 *mutation to 3R. We did identify a single deficiency in the Bloomington kit which fails to complement the *PL26 *lethality, but hemizygotes of *PL26 *with this deficiency do not show the spindle misalignment phenotype (not shown), and we have been unable to map the *PL26 *phenotype further.

#### PL17

One mutant, *PL17*, was initially identified in our pupal lethal screen as having a high mitotic index but overall reduction in the size of homozygous larval brains, suggesting a defect in cell cycle progression (described below). In addition to these defects we observed a frequent mislocalization of Miranda to discrete cytoplasmic regions, although localization of the apical marker Inscuteable appears unaffected (Figure 2D, D'). Co-staining with the centrosomal markers Centrosomin (Cnn) or γ-tubulin (not shown) suggests that in these neuroblasts Miranda is located in a pericentrosomal region during mitosis. This phenotype is incompletely penetrant, and some neuroblasts exhibit normal Miranda crescents, while others have both crescents and pericentrosomal Miranda, presumably a reflection of differences in the perdurance of maternally provided protein between cells. MARCM clones of *PL17 *appear phenotypically wild-type (not shown), and we interpret this as a consequence of the perdurance of wild-type protein within clones. Deficiency mapping and complementation testing revealed that *PL17 *is allelic to *imaginal discs arrested (ida)*, which encodes the *Drosophila *APC5 homologue, and a more detailed characterization of the defects observed in this line will be presented elsewhere (CS, PMO, R. Tuxworth and WC, manuscript in preparation).

#### J16

**Figure 3:**
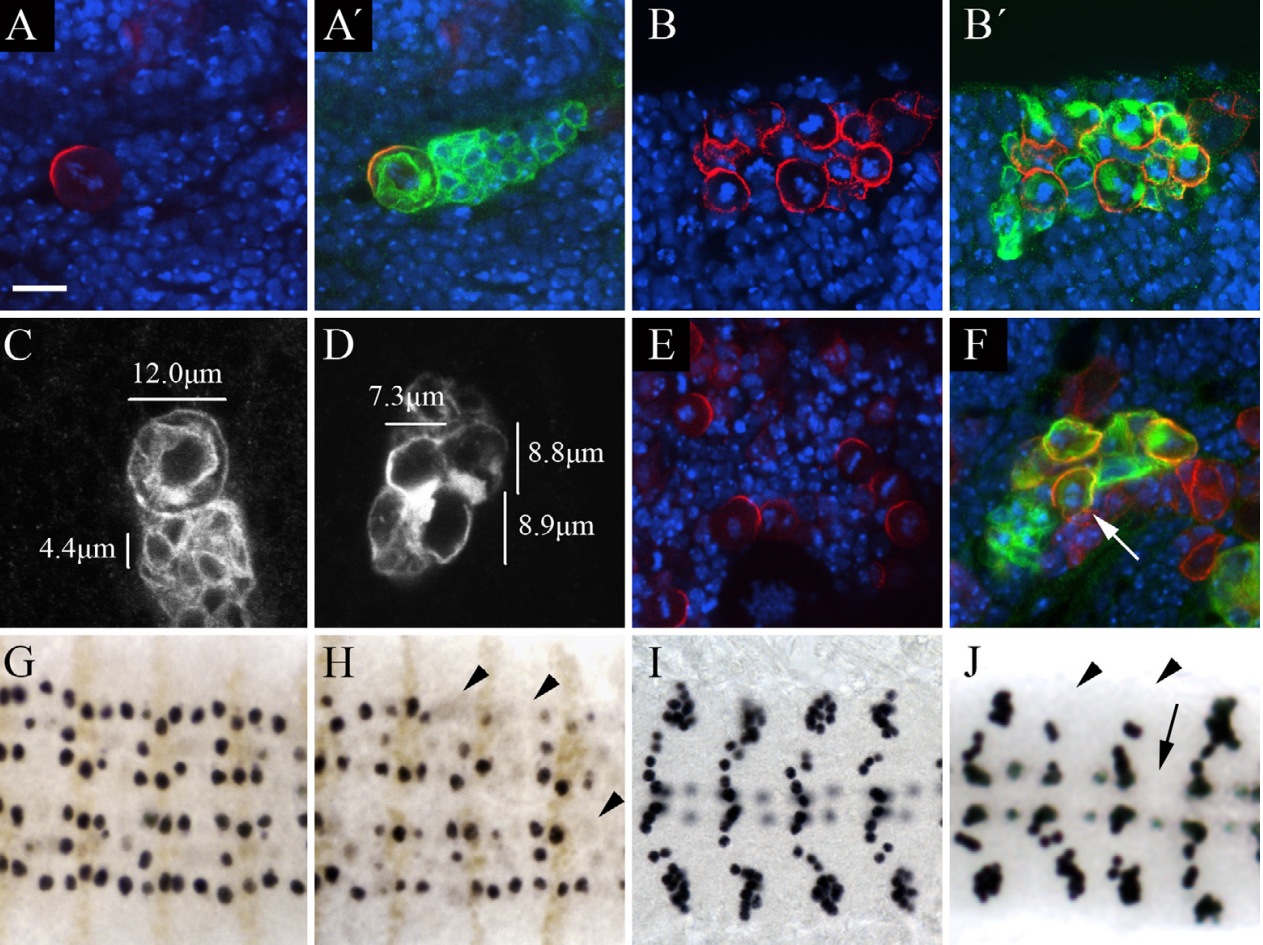
**The *J16 *mutation leads to defects in neuroblast cell fate**. (A, B) The size asymmetry of neuroblast divisions is disrupted in *J16 *clones. DNA staining (blue); Miranda (red); CD8::GFP (green, A', B'); Bar: 10 μm. Wild type clones (A) contain a single large neuroblast and a number of smaller progeny. Only the neuroblast and occasionally GMCs express Miranda. *J16 *clones (B) contain multiple cells expressing Miranda, all of which are of a similar size, and many of which appear to be arrested in metaphase. (C, D) Cells in *J16 *clones are intermediate in size. Neuroblasts in wild type clones visualised with anti-GFP staining (C) are approximately 12 μm in diameter, while neurons are approximately 4 μm across. In contrast, cells in *J16 *clones (D) are 7–9 μm in diameter. (E, F) The *J16 *chromosome carries an allele of *polo*, but this does not cause the cell size phenotype. DNA staining (blue); Miranda (red); CD8::GFP (green, F). *J16*/*Df(3L)Exel9636 *hemizygote brains (E) show the *polo *metaphase arrest phenotype. *J16 *clones generated in larvae carrying a *polo *genomic rescue construct (F) are not arrested in metaphase (arrow indicates cell in anaphase; note that Miranda does not correctly form a crescent in this cell) but still show the cell size phenotype. (G, H) Loss of neuroblast marker expression in *J16 *embryos. Flat preparations of stage 8–9 wild type (G) and homozygous *J16 *(H) embryos stained with anti-Worniu (black) and anti-Engrailed (brown). Worniu staining in wild type embryos reveals a stereotypical array of neuroblasts. In *J16 *embryos there is a loss of Worniu expression at a low frequency (arrowheads) suggesting a loss of neuroblast cell fate. (I, J) Loss of neuroblast progeny in *J16 *embryos. Flat preparations of stage 16 wild type (I) and *J16 *embryos (J) stained with anti-Eve. Eve is a marker for the progeny of four embryonic neuroblasts (see text). In *J16 *embryos, Eve staining is absent at a low frequency in the EL neurons (arrowheads), RP2 neurons (arrow) and CQ neurons (not shown).

We isolated a single mutant line, *J16*, which appears to have defects in the size asymmetry of neuroblast divisions within mutant clones. Wild type clones invariably contain a single large neuroblast accompanied by a number of much smaller GMCs and neurons. Miranda expression is found in neuroblasts, and occasionally in GMCs, but never in post-mitotic neurons (Figure 3A). In contrast to the wild type situation, clones of the *J16 *chromosome contain a number of large cells, many of which express Miranda, suggesting that the progeny of *J16 *neuroblast divisions fail to adopt the correct cell fate (Figure 3B). Examining wild type clones we find that the neuroblasts are typically around 12 μm in diameter, with the neurons roughly 4 μm across. Cells within *J16 *clones are intermediate in size to that of neuroblasts and neurons, with sizes in the region of 7–9 μm (Figure 3C, D).

The majority of cells in *J16 *clones appear to be in metaphase, with Miranda localized to a cortical crescent, indicating a cell cycle arrest in *J16 *clones. Deficiency mapping using the Bloomington kit revealed a single lethal region containing the *polo *locus and subsequent complementation tests revealed that the *J16 *chromosome carries an allele of *polo*. Hemizygotes of *J16 *with a deficiency for the *polo *region (*Df(3L)Exel9636*) survive to the third larval instar, and brains of these animals contain appear to have a high rate of metaphase arrest in neuroblasts (Figure 3E), but do not exhibit any phenotype suggesting a defect in asymmetric neuroblast division. This observation suggests that a second mutation in the *J16 *line is responsible for the cell size defects observed in clones.

To rule out any contribution of *polo *to the cell size phenotype observed in *J16 *clones, we introduced a *polo *genomic rescue fragment carried on the second chromosome into the *J16 *mutant background [42]. Clones of *J16 *with *polo *function thus restored do not exhibit the metaphase arrest phenotype, but still contain multiple similarly sized Miranda-expressing cells (Figure 3F; arrow indicates a cell in anaphase). We have not undertaken a detailed analysis of asymmetric cell division in these clones, and it is not yet clear whether the phenotype is a reflection of a symmetric mode of division such as has been described in early larvae [32].

To investigate the consequence of these defects in neuroblast division we examined the expression of neuroblast and cell fate markers in homozygous *J16 *embryos. We do not see any defects in neurogenesis in *J16 *embryos, as assayed by the expression of the proneural marker Achaete first in proneural clusters and then neuroblasts (not shown). Although neuroblasts form correctly in *J16*, by stage 9 we observe a loss of expression of Worniu, a marker for all embryonic neuroblasts [28,43,44], at a low frequency (Figure 3G, H). To determine whether this apparent loss of neuroblast identity affects the specification of cell fate in neuroblast progeny cells, we examined the expression of the neuronal marker Even-skipped (Eve) in stage 16 *J16 *embryos (Figure 3I, J). In wild type embryos, Eve is expressed in ~ 20 neurons per hemisegment: the aCC/pCC, CQ, and RP2 neurons, and the EL neuron cluster, which are the respective progeny of four neuroblasts [45]. As the early loss of neuroblast identity would suggest, we find a loss of Eve-expressing neurons with a low frequency (~ 2% for CQ and RP2 neurons, 4% for EL neurons, n = 420). In all cases the entire progeny of an individual neuroblast are lost, suggesting that neuroblasts which lose Worniu expression in *J16 *embryos do not give rise to any of the appropriate progeny. As in larvae, introduction of a *polo *genomic rescue fragment was unable to rescue this *J16 *phenotype (not shown). The low penetrance of neuroblast defects in the embryo suggests the perdurance of maternal protein may be masking the embryonic phenotype. As we do not obtain fertilized eggs in *J16 *germline clones, even with the restoration of *polo *function, we have not explored this further.

The *J16 *mutation appears to lie outside the region uncovered by the Bloomington deficiency kit, and further investigation will be required to identify the genomic region responsible for these defects.

#### D76

**Figure 4:**
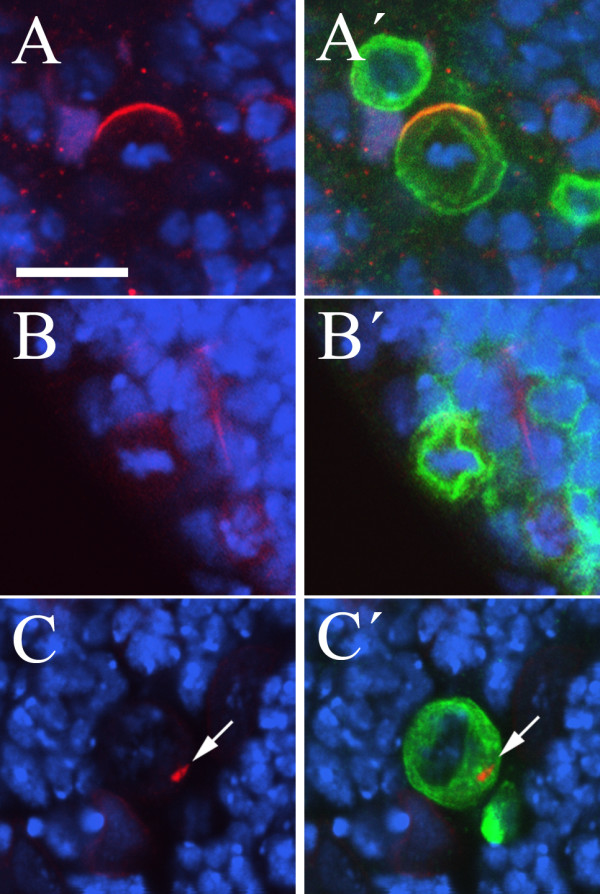
**The *D76 *mutation affects cell proliferation and Miranda localization**. (A-C) Miranda but not Inscuteable crescents are lost in *D76 *clone neuroblasts. DNA staining (blue); Inscuteable (red, A, A'); Miranda (red, B-C'); CD8::GFP (green, A', B', C'); Bar: 10 μm. Although a crescent of Inscuteable (A, A') forms correctly in *D76 *neuroblast clones, Miranda crescents are never observed (B, B', C, C'); occasionally Miranda localization to spots in the cytoplasm is observed (arrows in C, C').

We isolated a single allele, *D76*, in which dividing neuroblasts are almost never seen. On examining large numbers of clones we were able to determine that the rare neuroblasts which enter metaphase do not form a basal Miranda crescent, although Miranda is expressed and can be detected at low levels in the cytosol and Inscuteable is localized appropriately to the apical cortex (Figure 4A, A', B, B'). In some neuroblasts we observe punctate spots of Miranda staining in the cytoplasm (indicated by an arrow in Figure 4C), the identity of which is unclear. Deficiency mapping uncovered a single lethal deficiency from the Bloomington kit, and complementation testing indicated that this corresponds to a hit in the *trio *gene. However, the phenotype of *trio *mutant larvae has been described and appears unlikely to be responsible for the phenotypes we observe in neuroblasts. Recombination mapping using a *ru h th st cu sr e*^*s *^*ca *chromosome revealed that the phenotype maps proximal to *ru*, excluding *trio*, and distal to *th *(data not shown). However, we have been unable to identify additional lethal deficiencies within this interval, and so have not mapped the *D76 *mutation further.

### Cell division defects

In addition to the four lines with phenotypes during neuroblast asymmetric division, we identified a total of 76 mutations, in 46 complementation groups, which exhibit defects in cell division (Table 1: Cell division defects). We have categorized these as having defects in cell proliferation or chromosome separation, or as giving rise to multinucleate or metaphase arrested cells; a final category contains lines which have a variety of membrane or vesicular defects.

#### Proliferation defects

In our pupal lethal screen we identified three lines in which the brains of homozygous larvae appear reduced in size (Table 1: *Proliferation defects)*. Two of these mutations – *PL13 *and *LVC73 *– give similar phenotypes. The optic lobes of the brain are generally small, with fewer neuroblasts than wild type, and we rarely observe mitotic cells (Figure 2E, F). Staining with an antibody against phospho-histone H3 (anti-PH3) revealed that the proportion of dividing neuroblasts in these brains is greatly reduced compared to wild type (for example 22% of neuroblasts in the central brain region stain with anti-PH3 in *PL13 *homozygous brains (n = 37), compared to 45% in *PL13*/*FRT2A *heterozygotes (n = 60)), although when we do see neuroblasts in metaphase the localization of Miranda and orientation of the mitotic spindle appear normal. Despite their phenotypic similarities, these mutant lines do not show any lethality as transheterozygotes and their lethal regions map to distinct regions of the chromosome.

An additional line, *PL17 *– the Miranda localization phenotype of which is described above – gives larvae with brains in which the optic lobes are again reduced in size. However, the proportion of dividing neuroblasts in this line is significantly increased compared to wild type (76% of neuroblasts stain with anti-PH3 (n = 45)), suggesting a delay at meta- or anaphase (Figure 2G). We identified two overlapping deficiencies in the Bloomington kit, *Df(3L)HR119 *and *Df(3L)GN34*, which fail to complement the lethality of *PL17*, and complementation testing revealed that *PL17 *is allelic to *imaginal discs arrested*.

**Figure 5:**
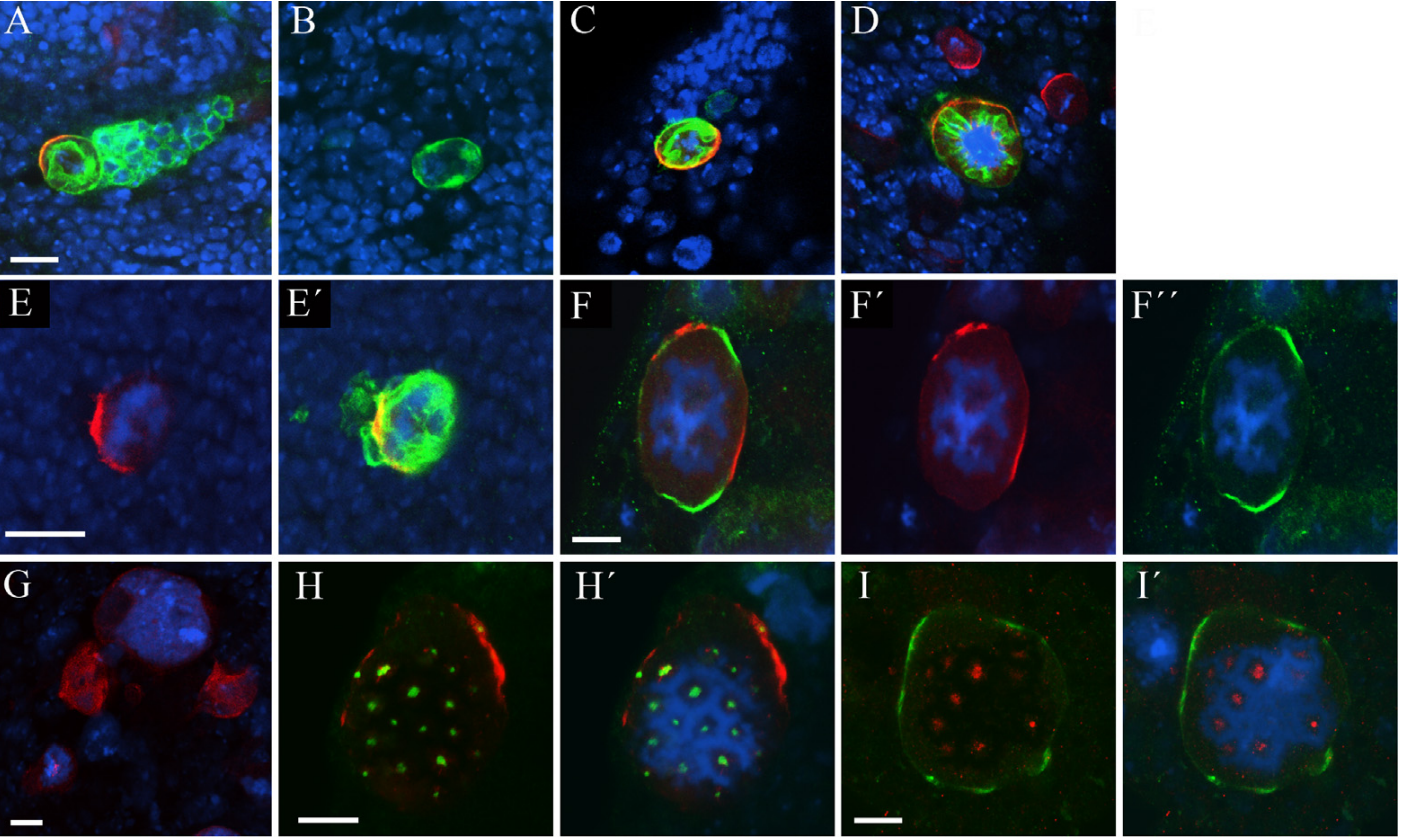
**Mutations affecting neuroblast proliferation and chromosome separation**. (A-D) Mutations affecting cell proliferation. DNA staining (blue); Miranda (red); CD8::GFP (green); Bar: 10 μm. Wild type clones (A) contain a single large neuroblast and a number of smaller progeny. *B18 *clones (B) contain fewer progeny cells and we rarely observe mitotic neuroblasts. In clones of *E45*, which is allelic to *makos*, we also see few progeny but neuroblasts have strong crescents of Miranda and appear arrested in a metaphase-like state, although some separation of sister chromatids is observed (C). (D-I) Mutations affecting chromosome separation. DNA staining (blue); Miranda (red, D-H'); CD8::GFP (green, D, E'); Inscuteable (green, F, F", I, I'); Centrosomin (green, H, H'); γ-tubulin (red, I, I'); Bar: 10 μm. Both *DL42 *(D) and *C93 *(E, E') clones contain large cells with a high DNA content which never appear to divide. *C93 *is allelic to *separase*, and *C93*/*Df(3L)Exel6106 *hemizygote (F-F'') or *C93*/*sse*^*M13 *^transheterozygote (G) neuroblasts are large with a high DNA content. In hemizygous neuroblasts we observe multiple crescents of Miranda (F, F') and Inscuteable (F, F''), which never overlap. Labelling with anti-Centrosomin (H, H') or γ-tubulin (I, I') reveals that *C93 *hemizygous neuroblasts contain multiple centrosomes.

In our clonal screen, we isolated nine mutations in which we see clones of only one or two cells but in which the neuroblasts do not show any gross morphological defects. Because of the small numbers of mutant clone neuroblasts generated in our screening regimen we have not attempted to accurately measure the mitotic index in these mutations, but in eight out of nine cases the lack of mitotic neuroblasts observed suggests that the mitotic index is greatly reduced (Figure 5A, B).

These eight mutations fall into five complemention groups which we have mapped to separate regions of 3L (Table 1: *Proliferation defects*). Testing known mutants in these regions revealed that the two mutations that fall into complementation group 7 are allelic to *small-minded (smid)*. Furthermore, the phenotype observed in clones of these mutations reflects the described phenotype of *smid *homozygous larvae in which proliferation of postembryonic neuroblasts is reduced [46]. As described above, one of these complementation groups, *D76*, also gives rise defects in Miranda localization in the rare metaphase neuroblasts observed.

In addition to these lines with very low mitotic indices, we identified one line, *E45*, in which clone neuroblasts appear to be arrested in a metaphase-like state with condensed chromosomes, although without the formation of a clear metaphase plate (Figure 5C). This mutation mapped to a region containing *makos*, the *Drosophila cdc27 *homologue, alleles of which are known to give a similar metaphase-like arrest phenotype [47], and testing against a known mutation indicated that our line is a new *mks *allele. Sequencing of the *mks *locus in our stock revealed a single nucleotide G→A transversion leading to the conversion of a tryptophan residue at amino acid 622 – before the conserved TPR repeat region – into a stop codon, suggesting that our mutation represents a loss of function allele.

#### Chromosome separation defects

A number of mutations were isolated which appear to show defects in sister chromatid separation at anaphase (Table 1: *Chromosome separation defects*). These three complementation groups give rise to clone neuroblasts which appear to have an abnormally high DNA content but which do not contain multiple nuclei (Fig 5D, E, E'). Complementation testing with known cell division mutants on 3L identified one of these complementation groups, containing five mutations, as allelic to *Kinesin-like protein at 61F *(*Klp61F*; Figure 5D). A further group, with a single member *C93 *(Figure 5E, E'), mapped to a deficiency containing *separase *(*sse*), previously known to be required for chromosome separation, and sequence analysis indicated that the *C93 *chromosome carries a G→A transversion at the exon 6 splice acceptor of *sse*, presumably leading to the formation of a truncated protein. Transheterozygotes of *C93 *with *sse*^*M13 *^or hemizygotes with *Df(3L)Exel6106*, which uncovers the *sse *locus, survive until late third instar stages and show phenotypes similar to those observed in C93 clones. These brains contain large cells which have an increased DNA content (Figure 5F, F', F", G): we note that the phenotype in these animals is more severe than we observe in clones, suggesting – as we observed for the *PL17 *mutation – that these clones retain some wild-type *sse *protein.

Although no obvious Miranda localization defects in *C93 *clones were observed, we found that the large neuroblasts in *C93*/*Df(3L)Exel6106 *hemizygotes frequently contained several cortical crescents of Miranda instead of a single basal crescent. Interestingly, when we examined the localization of Inscuteable in these larvae, we found that neuroblasts with several Miranda crescents also had several Inscuteable crescents, and that these never overlapped (Figure 5F, F', F"). In wild type neuroblasts the domains of Inscuteable and Miranda never abut precisely but are separated by a region of cortex containing neither protein, and in these mutant neuroblasts we see a similar region between the Inscuteable and Miranda crescents. To investigate the origin of the multiple crescents in *C93 *hemizygotes we stained neuroblasts with the centrosomal markers Centrosomin (Cnn) and γ-tubulin (Figure 5H, H', I, I'), and found that these cells contain large numbers of centrosomes; presumably the failure to separate sister chromatids at anaphase leads to multiple rounds of centrosome and DNA duplication and failed cell division. It is possible that the presence of multiple centrosomes causes the formation of multiple crescents of Inscuteable, by a mechanism similar to that described for the centrosome-induced cortical polarity of the *C. elegans *embryo [48], although we have not attempted to establish a direct correspondence between individual centrosomes and Inscuteable crescents in these cells. The mechanism responsible for the basal positioning of Miranda in wild type neuroblasts could then lead to the formation of Miranda crescents in those regions of the cortex not occupied by Inscuteable.

#### Multinucleate cells

Two lines isolated in our pupal lethal screen, *IV61 *and *CMV111*, are allelic to one another and have a phenotype in larval brains consistent with a defect in cytokinesis: we frequently observe large cells which appear to have an unusually high DNA content (Figure 2H). Complementation testing with known cell division mutants on chromosome 3 indicates that this complementation group is allelic to *sticky*, which encodes a serine/threonine kinase related to the mammalian citron kinase, and which has previously been shown to be required for cytokinesis in all *Drosophila *tissues [49].

**Figure 6:**
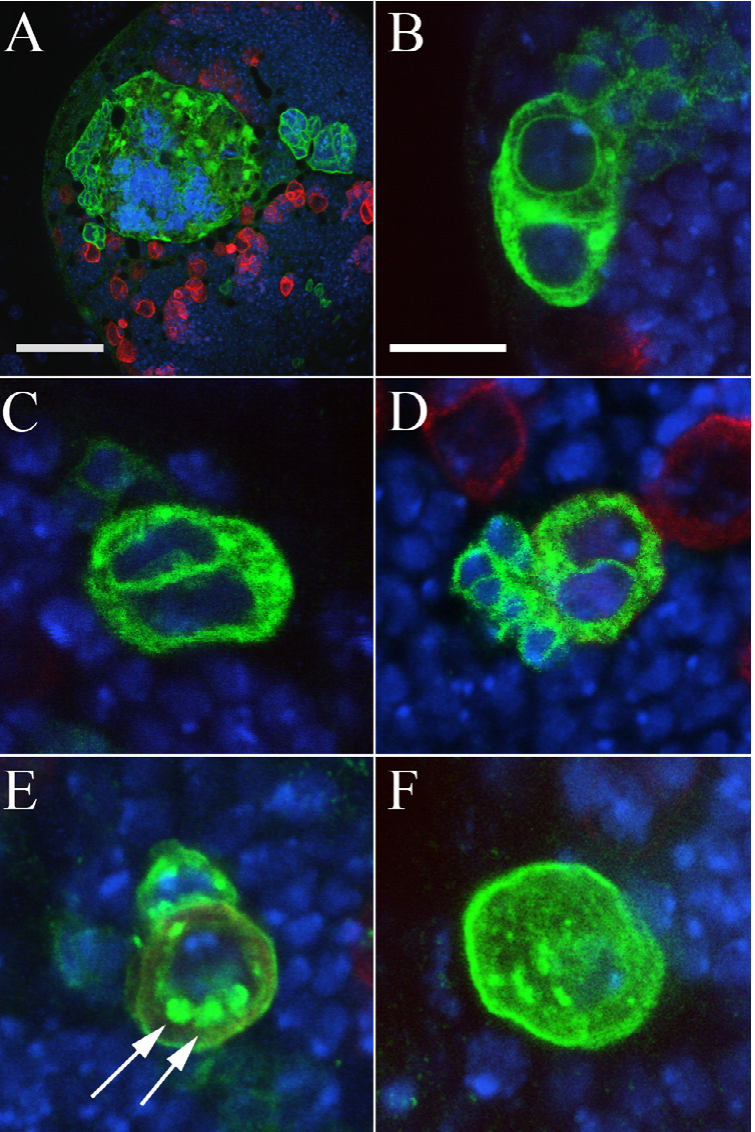
**Multinucleate cells in mutant clones**. (A-F) Mutations leading to cytokinesis or membrane defects in MARCM clones. DNA staining (blue); Miranda (red); CD8::GFP (green); Bar: 50 μm (A), 10 μm (B-F). The *C33 *mutation, which maps to the *pebble *locus, result in a dramatic increase in cell size and DNA content and clones can occupy a substantial proportion of the optic lobe (A). Clones of the *J2 *(B), *D24 *(C) or *GL45 *(D) chromosomes also give rise to multinucleated cells which typically contain only two nuclei. Mutation of *O29 *(E) or *CMV45 *(F) leads to the formation of punctae labelled with CD8::GFP (arrows) or an excess of GFP staining; the origin of both these phenotypes is unclear.

In the clonal screen we identified a further allele of *sti*, *D97 *– which has a similar phenotype to the pupal lethal alleles – as well as 24 additional mutations, in 15 complementation groups, which also give rise to multinucleate neuroblasts (Table 1: *Multinucleate cells*). One of these mutations, *C33*, shows a severe cell division phenotype in neuroblasts and was mapped to the *pebble *locus, previously shown to be required for cytokinesis ([50]; Figure 6A). Mutant neuroblasts from the other 14 complementation groups typically contain two to four nuclei and may also represent a failure to complete cytokinesis (Figure 6B, C, D). One of these is allelic to *TBP-associated factor 4 *(*Taf4*), and the multinuclear phenotype is presumably a downstream consequence of defects at the transcriptional level. The remaining complementation groups mapped to a number of chromosomal regions with the exception of groups 30 and 31 which complement the entire Bloomington deficiency kit and were not mapped further.

#### Membrane/vesicular defects

The final group of mutants we classified as having cell division phenotypes are those which give rise to small clones, typically of one or a few cells, in which the most striking defects are associated with the CD8::GFP which labels membranes (Table 1: *Vesicular/membrane defects*). Whereas in wild type neuroblast clones we observe the CD8::GFP outlining the cell and nucleus, and surrounding the mitotic spindle, in a number of these lines we frequently see multiple brightly labelled punctae within the cell, the identity of which is unclear (Figure 6E, arrows). In other lines in this category we observe the CD8::GFP staining to fill the majority of the cell, suggesting an excess of membrane is present, but without necessarily concentrating into bright spots (Figure 6F). In total we isolated 31 alleles of 18 genes, five of which we have identified by deficiency mapping and complementation analysis. We have isolated novel alleles of *reptin*, *Taf-6*, *Int6 *and *Aats-ile*, which all encode proteins involved in general cellular processes at the level of transcription or translation. The phenotypes observed in mutant clones of these genes presumably reflect pleiotropic effects resulting from a disruption of these basal processes. We have also isolated a new allele of *neurexin *which is required for correct vesicle trafficking at the neuromuscular junction [51]. The remaining mutants in this category may therefore disrupt genes whose functions are required for membrane biosynthesis and/or vesicle targeting.

## Discussion

We found a total of five complementation groups that affect the distribution of Miranda in dividing neuroblasts or the size asymmetry of the neuroblast division. At the time of writing, two have been mapped to a gene. One of these is *separase *which seems unlikely to be involved directly in the mechanism of asymmetric cell division. The second is *ida *which appears to disrupt the basal localization of Miranda leading to an accumulation of Miranda in a pericentrosomal compartment. A more detailed description of this phenotype, which suggests a novel connection between the APC/C and the localization of cell fate determinants, will be presented elsewhere (CS, PMO, R. Tuxworth and WC, manuscript in preparation). Several of the other mutations isolated in the screen are also likely to have a direct influence on the asymmetry machinery. In particular, we have identified a mutation affecting metaphase spindle orientation in neuroblasts, a process which is critical to the correct segregation of cell fate determinants, and another which appears to perturb the size asymmetry of the neuroblast division and the correct establishment of different identities in daughter cells.

We did not find as many asymmetric division mutations as we might have expected given the scale of the screen: we estimate, using a Poisson approximation, that we have achieved approximately 80% saturation of 3L. Although it is possible that the design of our screen has prevented the detection of some genes, we believe that this is a reflection of the rather small number of genes involved exclusively in asymmetric cell division. Indeed, a similar screen of 3R has detected new alleles of several known players of neuroblast asymmetric division including *miranda, prospero *and *scribbled*, a tumour suppressor in the Lgl/Dlg pathway (RS-N, WGS and WC, unpublished data). This suggests that our screen methodology is effective at finding genes involved in neuroblast asymmetric division, and that it can in the future be applied to the whole genome to find further novel components of this machinery. The major limitation with the approach at present is that it is rather labour intensive, which necessarily leads to a low throughput compared to other screening methods. The use of live imaging methods, coupled with the GFP and RFP fusion reagents widely in use, would most likely relieve this bottleneck and allow a higher throughput. Similarly, improvements to the crossing schemes could be made. If chromosomes could be screened without the need to establish stocks of each one, perhaps with mutations being recovered from the siblings of the larvae examined, an F1 screen could be conducted which would enable a genome-wide saturating screen to be carried out much more rapidly.

In addition to finding several new polarity genes, we have isolated large numbers of mutations affecting cell division, and this seems to be a particular strength of such a clonal approach. Looking only at complementation groups which give rise to multinucleate neuroblasts we have found mutations in 13 regions not previously known to be involved in cytokinesis, and similar screens may prove beneficial to laboratories with a specific interest in aspects of cell division. As with other screening methods, in several cases mapping of the gene responsible for a cell division phenotype has yielded unexpected results: for example, mutations in *taf4*, involved in transcriptional initiation, appear to cause a failure in cytokinesis. Nonetheless, we have identified alleles of a number of previously reported cell division genes and we anticipate that a substantial number of the mutations described here will be directly involved in the processes of cell division.

Early studies of factors involved in asymmetric cell division described a number of genes with phenotypes that specifically disrupt this process and for which the establishment of neuroblast polarity is the primary or only function. It is now starting to become clear, however, that many of the molecules required for neuroblast asymmetry are also employed in a number of other roles within the cell, as well as in a range of tissues during development. For example, *lethal giant larvae (lgl) *and *discs large (dlg)*, involved in the localization of basal components in neuroblasts, are both tumour suppressors, and in their absence larval brains and discs show a dramatic overgrowth (reviewed in [52]). Similarly, at least two Myosins, necessary for a range of cellular processes throughout development, are involved in Miranda localization [9,12]. This is probably why simple zygotic genetic screens looking for defects specifically in neuroblast asymmetric division are nearing their limit. In our clonal screen we found several mutations which adversely influence neuroblast proliferation but also disrupt the formation of the basal Miranda crescent. The molecules responsible for these phenotypes remain to be isolated, but may shed light on the connection between the cell cycle and localization of cell fate determinants. The differences in phenotypes observed in these lines will be of particular interest. For example, in *D76 *the Miranda crescent is entirely lost, while in *PL17*, affecting the *Drosophila *APC5 homologue, Miranda is observed to be strongly associated with the centrosome – the latter case is intriguing as Miranda has previously been found to be centrosomally localized in a cell cycle dependent manner, although how this relates to Miranda localization and function is unclear [19,53].

Aside from the clear utility of the MARCM system in screening for mutations affecting aspects of cell division, we consider that it could easily be adapted for use in a mis-expression screen. As clones are positively labelled by *elavGal4 *directing expression of GFP, the presence of an EP insertion [54], or one of the related UAS-containing transposable elements, not necessarily on the chromosome arm carrying the *FRT *site, would lead to transcription of genes downstream of this element specifically in labelled clones, and circumvent any early lethality caused by ectopic expression, a frequent limitation of such screens.

## Conclusion

Previous screens for components of the asymmetric division machinery have focused on embryonic phenotypes, and are now reaching saturation. Here we have used a clonal approach to screen neuroblasts in the third instar larval brain, and have identified several novel mutations, the identification and further study of which will advance our understanding of the process of neuroblast asymmetric division.

## Methods

### Fly stocks and genetics

All *Drosophila *stocks were reared and maintained on standard yeast-cornmeal-agar medium [55] and all experiments were performed at 25°. To generate mutant lines, *w *flies carrying an *FRT *element inserted at polytene segment 79D-F (*FRT 2A*) were first isogenized for the third chromosome. Three to five day old males were then mutagenized by feeding with 1% sucrose solution containing 25 or 38 mM EMS as described previously [56]. Mutagenized males were crossed *en masse *to virgin females of the genotype *TM8/TM6B, Tb, Ubi-GFP*. Single male progeny were crossed to the balancer stock to establish stocks in which the mutagenized third chromosomes were balanced over *TM6B, Tb, Ubi-GFP. *Lines in which the mutagenized chromosome was homozygous viable were discarded.

Stocks in which animals homozygous for the mutagenized *FRT2A *chromosome were viable at the wandering third larval instar stage (350 lines), as assayed by the absence of the *Tb *marker, were screened by antibody staining of mutant brains. Lines lethal before the third instar (1923 lines) were screened using the MARCM system [34]. Females of each mutant stock were crossed to males of the MARCM driver line *elavGAL4*^*C155*^,*hsp70Flp*/Y; UAS-*CD8::GFP*, UAS-*LacZ*; *tub-GAL80*, *FRT2A *(a gift from A. Gould and B. Bello). Crosses set up using females from the MARCM driver line and males from a mutant stock were significantly less productive, and these crosses were avoided except in cases in which few females of a mutant stock could be obtained.

After 24 hour periods of egg laying, progeny were heat-shocked twice for 2 hours in a 37° water bath at first instar and second instar stages to induce mitotic recombination. Brains of female non-*Tb *wandering third instar larvae were dissected and screened by antibody staining.

Following screening, lines were placed in complementation groups by pairwise complementation testing and tested against alleles of the following candidate genes on 3L: *pebble*, *encore*, *nuclear fallout*, *four wheel drive*, *pavarotti*, *fumble*, *polo*, *sticky *and *kinesin-like protein at 61F (Klp61F)*. This allowed us to identify alleles of *pebble*, *polo*, *sticky *and *Klp61F*. The remaining complementation groups were mapped initially by crossing to the third chromosome deficiency kit, provided by the Bloomington *Drosophila *stock centre. We performed further fine scale mapping with smaller deficiencies obtained from the Drosdel and Exelixis collections [57,58], as well as other deficiencies obtained from Bloomington, to define the minimal region containing each complementation group – deficiency breakpoints are described in Flybase [59]. Testing candidate genes in these regions allowed us to assign additional groups as *small-minded*, *TBP-associated factor 4 *and *-6 *(*Taf4 *and *-6*), *reptin*, *Isoleucyl-tRNA synthetase *(*Aats-ile*), *separase*, *makos*, *Int6 *and *neurexin*. A number of complementation groups containing only a single allele failed to complement more than one region of 3L. In several of these cases we were able to place the phenotype in a single region by examination of hemizygous phenotypes or by meiotic recombination with appropriate markers from a *ru h th st cu sr e*^*s *^*ca *chromosome followed by clonal analysis. Several groups complemented the entire Bloomington deficiency kit for 3L and were not mapped further.

Stocks, other than those used to test candidate genes and available from the Bloomington or Szeged stock centres, were *mks*^*1 *^[47], *P{GFP-polo} *[42] and *sse*^*M13 *^[60].

### Immunohistochemistry

Brains of wandering third instar larvae were dissected in 100 mM Na_2_HPO_4_/NaH_2_PO_4 _(PBS) and fixed in 4% formaldehyde (Polysciences) in PBS for 20 min at room temperature. Brains were washed in PBT (PBS + 0.1% Triton X-100) for 2 × 30 min, blocked in 5% normal goat serum in PBT for 30 min, incubated with primary antibody at 4° overnight, washed 3 × 20 min in PBT, incubated with secondary antibody for 2 hours at room temperature, washed 3 × 20 min in PBT, further dissected and mounted in Vectashield (Vector Laboratories). Antibodies used were mouse anti-Miranda [40], rabbit and mouse anti-GFP (1:1000 and 1:100, both Molecular Probes), rabbit anti-Phospho-Histone H3 (Upstate Biotechnology), rabbit anti-Inscuteable (1:1000 [61]), rabbit anti-Cnn (1:500 [62]), rabbit anti-Even-skipped (1:2000 [45]), mouse anti-Worniu (1:1000 [28]) and mouse anti-gamma tubulin (1:500, Sigma-Aldrich), together with monoclonal mouse anti-Engrailed/Invected [63], anti-Achaete [64] and anti-Prospero [20] obtained from the Developmental Studies Hybridoma Bank developed under the auspices of the National Institute of Child Health and Human Development (NICHD) and maintained by The University of Iowa, Department of Biological Sciences, Iowa City, IA 52242.

Secondary antibodies were obtained from Jackson labs (Cy3/HRP) or Molecular Probes (Alexa-488) and used at a concentration of 1:1000 (fluorescence) or 1:500 (HRP). DNA was visualized by the addition of ToPro-3-iodide (1:20,000, Molecular Probes) to one of the wash steps. Antibody staining of embryos was performed essentially as previously described [65]. Samples were viewed and images were taken using a Zeiss LSM 510 laser scanning confocal microscope or a Zeiss Axioplan 2 compound microscope. Images were processed using Adobe Photoshop.

## Authors' contributions

CS, WGS, WC and PMO designed the screen, which was carried out by CS, WGS and PMO with help from RS-N; CS and PMO performed deficiency mapping and further analysis of mutations; PMO wrote the manuscript with help from CS; all authors read and approved the manuscript.
